# Supplementary material for: Salicylic acid and RNA interference mediate antiviral immunity of plant stem cells
Source: Proc Natl Acad Sci U S A. 2023 Oct 12;120(42):e2302069120. doi: 10.1073/pnas.2302069120 (PMC10589665; doi:10.1073/pnas.2302069120)
Supplement: Supplementary file 1 — Appendix 01 (PDF) [file pnas.2302069120.sapp.pdf]

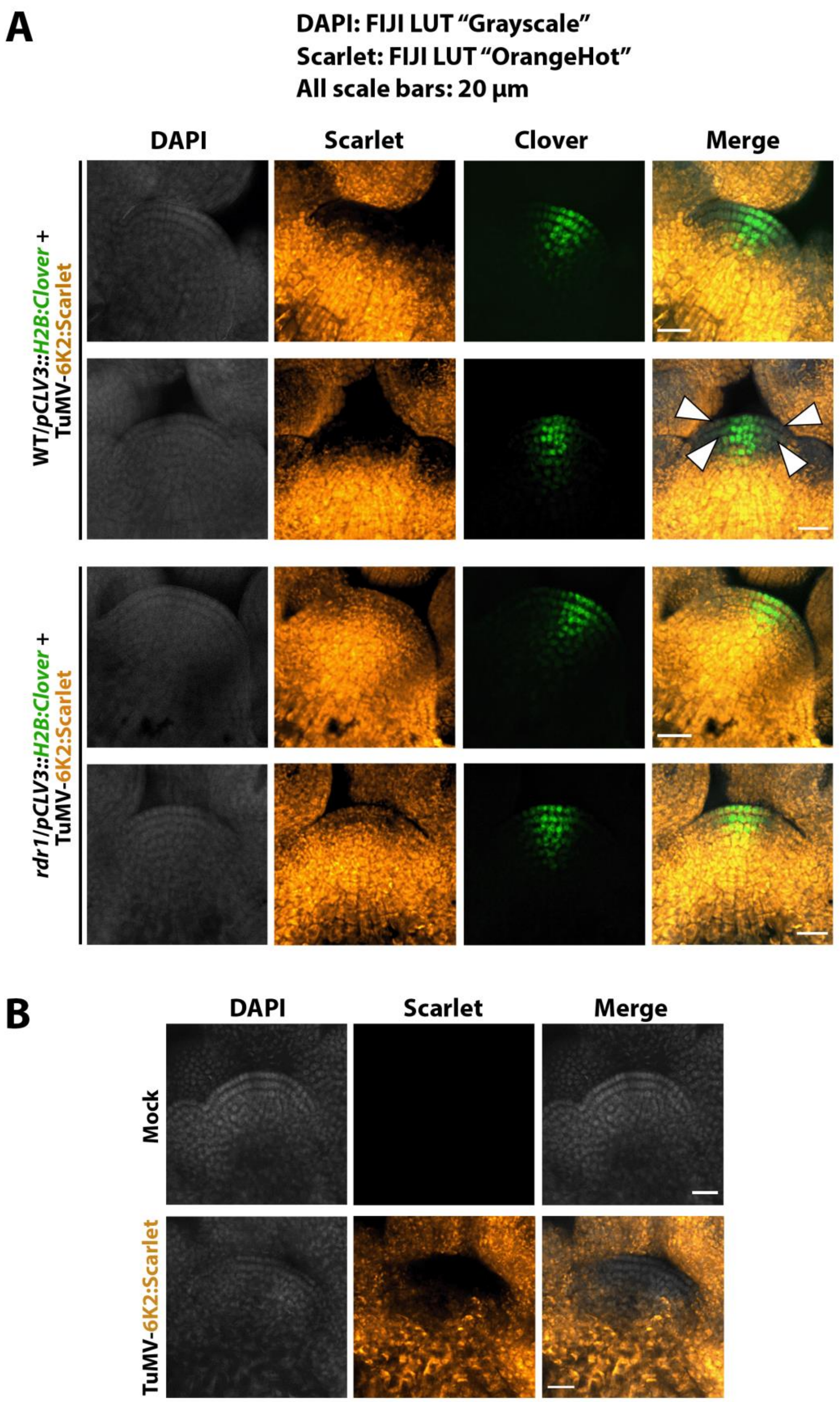

Figure S1: (A) Examples of main inflorescence shoot apical meristems (SAMs) from TuMV-6K2:Scarlet-infected WT and *rdr1* mutant transgenic lines expressing nuclear fluorescent reporter H2B:Clover in the stem cell domain of the SAM, through the *pCLV3* promoter, at 16-17 dpi. This color scheme is used for laser confocal microscopy images throughout this study: DAPI fluorescence is in grayscale, Clover is in green (except in Fig. 2D, E), Scarlet fluorescence is in orange-to-yellow (FIJI LUT "OrangeHot", with increasing signal intensity going from orange to yellow). Scale bars always indicate 20 µm. The white arrowheads delineate the "core" virus exclusion zone in WT plants, the two top-most cell layers: L1 and L2. **(B)** As in (A), but WT plants after mock-inoculation (top) or infection with TuMV-6K2:Scarlet.

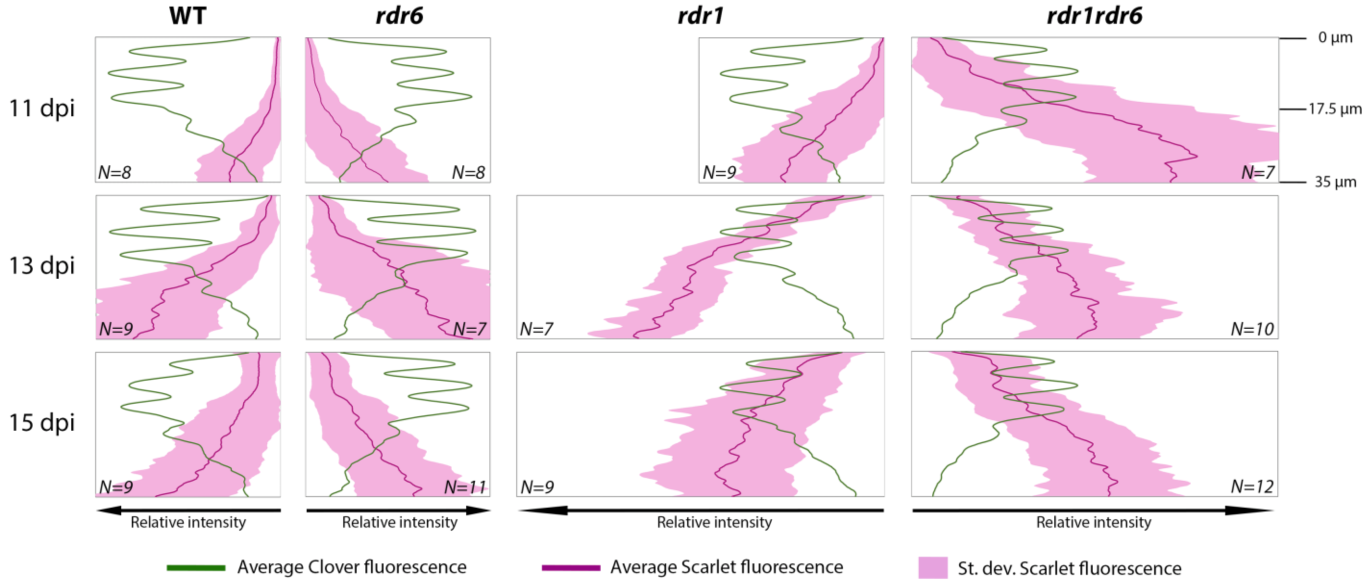

Figure S2: Plots showing fluorescence values over time, every 2 days from 11 to 15 days post-inoculation (dpi), in the top 35 µm of the SAM of (from left to right) WT, *rdr6*, *rdr1* and *rdr1rdr6* knock-out mutants (right). The green line indicates the average Clover fluorescence and identifies the L1-L2-L3 cell layers, the dark magenta line indicates the average Scarlet fluorescence (normalized in each sample to the respective Clover values), while the light magenta area indicates the standard deviation of Scarlet fluorescence among the samples. *N* indicates the number of shoot apical meristems analyzed.

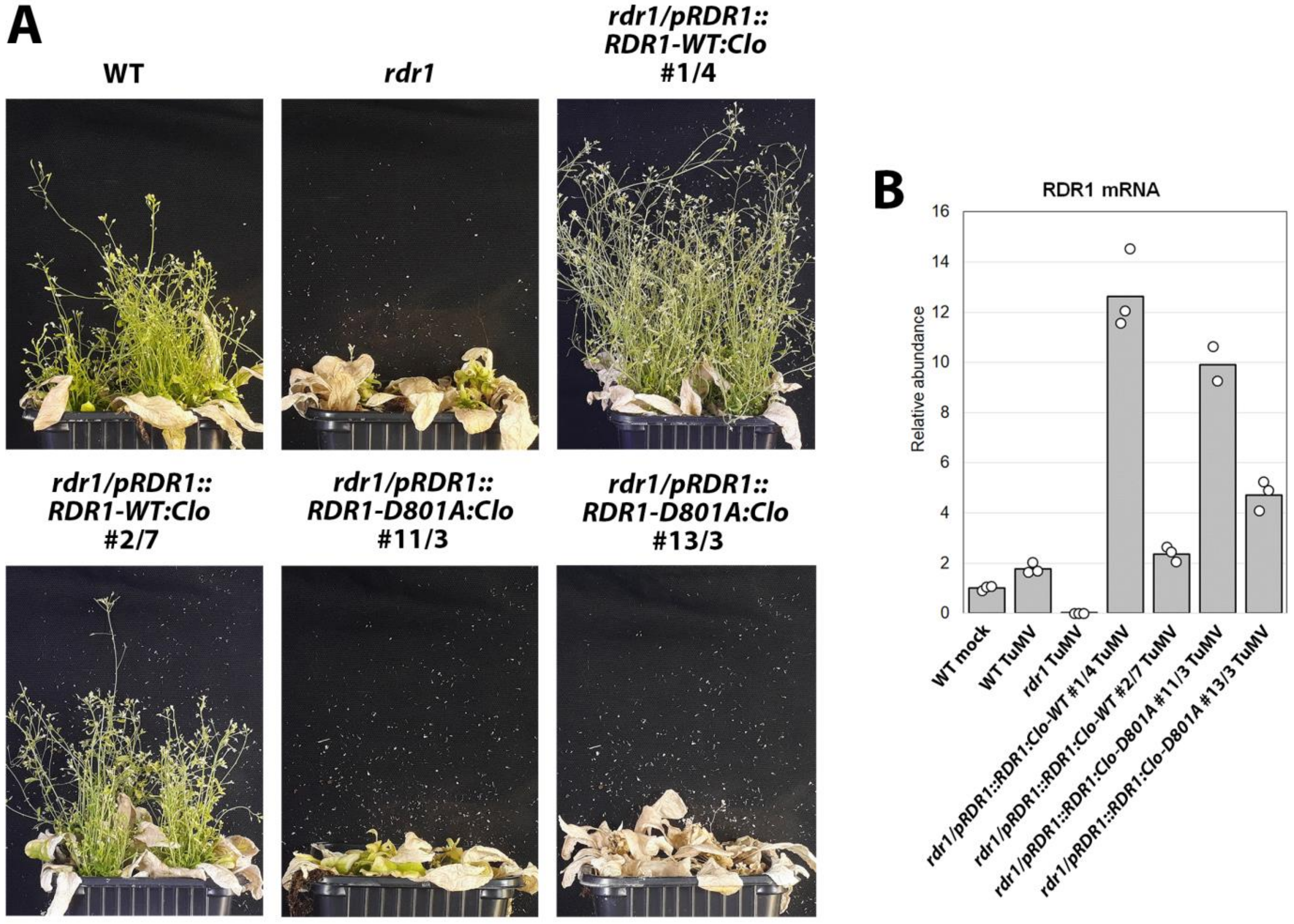

Figure S3: (A) Photos of plants one month after inoculation with TuMV-6K2:Scarlet. Top: all infected plants lose apical dominance, but WT generate fertile shoots from axillary meristems, while *rdr1* mutant plants do not. Bottom: *rdr1* mutants complemented with WT (left) or catalytically inactive D801A (right) alleles of Clover-tagged RDR1. Two independent transgenic lines per construct are shown. (B) RT-qPCR analysis to quantify RDR1 mRNA in the RDR1 complementation lines indicated. The primers used span the T-DNA insertion in rdr1-1. Dots indicate technical replicates.

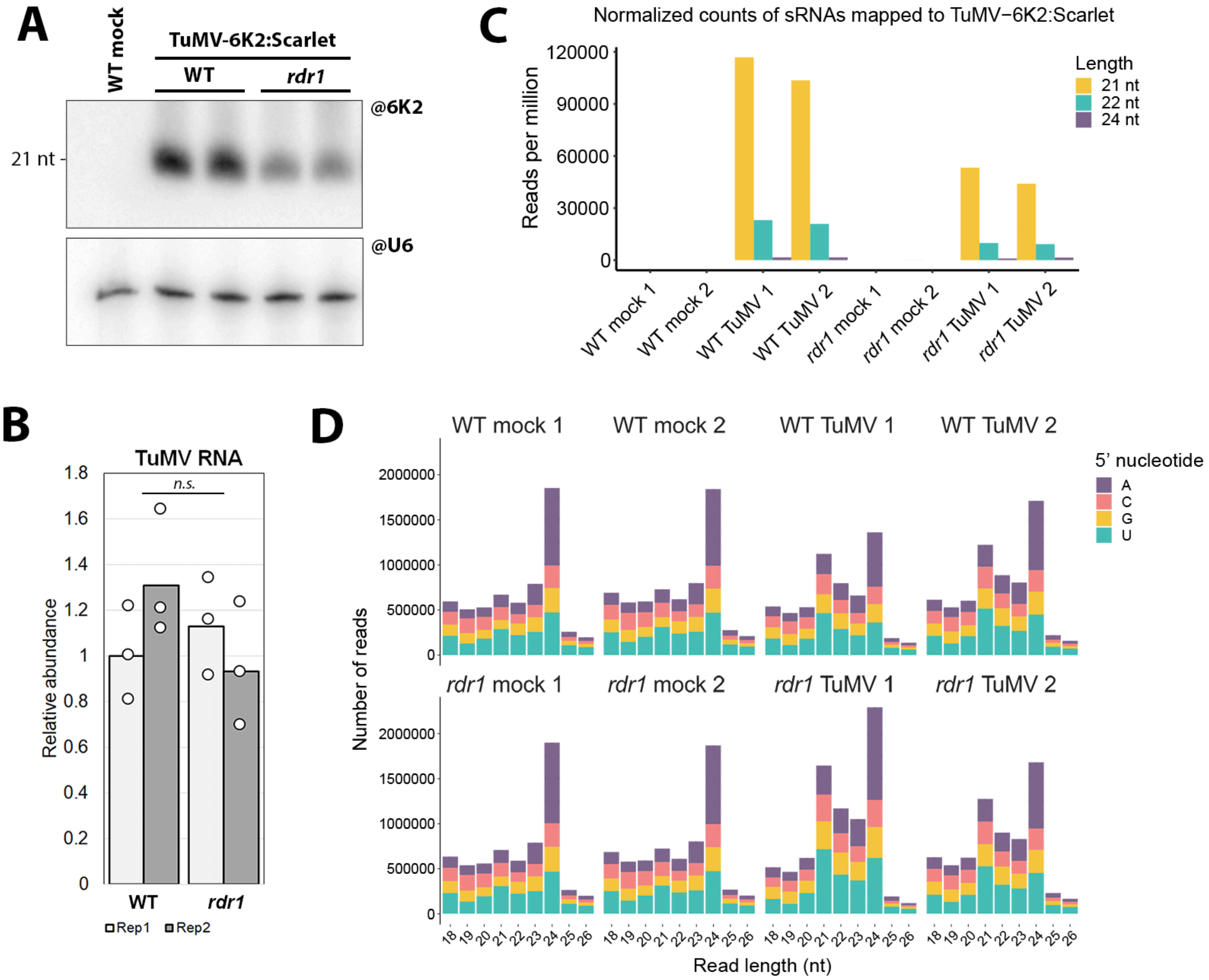

Figure S4: (A) PAGE northern blot detection of TuMV 6K2-derived sRNA in systemically infected WT and *rdr1* plants, 11 dpi. Biological duplicates are shown, each sample being a pool of tissue from 4-5 plants. Mock-treated control (plants inoculated with buffer only) is on the left, snRNA U6 is used as loading control. **(B)** RT-qPCR analysis to quantify TuMV gRNA in the samples described in (A). Each column is a biological replicate, each dot a technical replicate. *n.s.*: $p > 0.05$ **(C)** Bar plots showing the number of reads-per-million (divided into 21, 22 and 24 nt in length) of sRNA mapping to TuMV-6K2:Scarlet, as assessed by sRNA sequencing on duplicates of mock- and TuMV-infected apices (meristem and small flower buds) of WT and *rdr1* plants. **(D)** Size distribution (18 to 26 nt length) and 5' nucleotide of the total reads in the experiment described in (C).

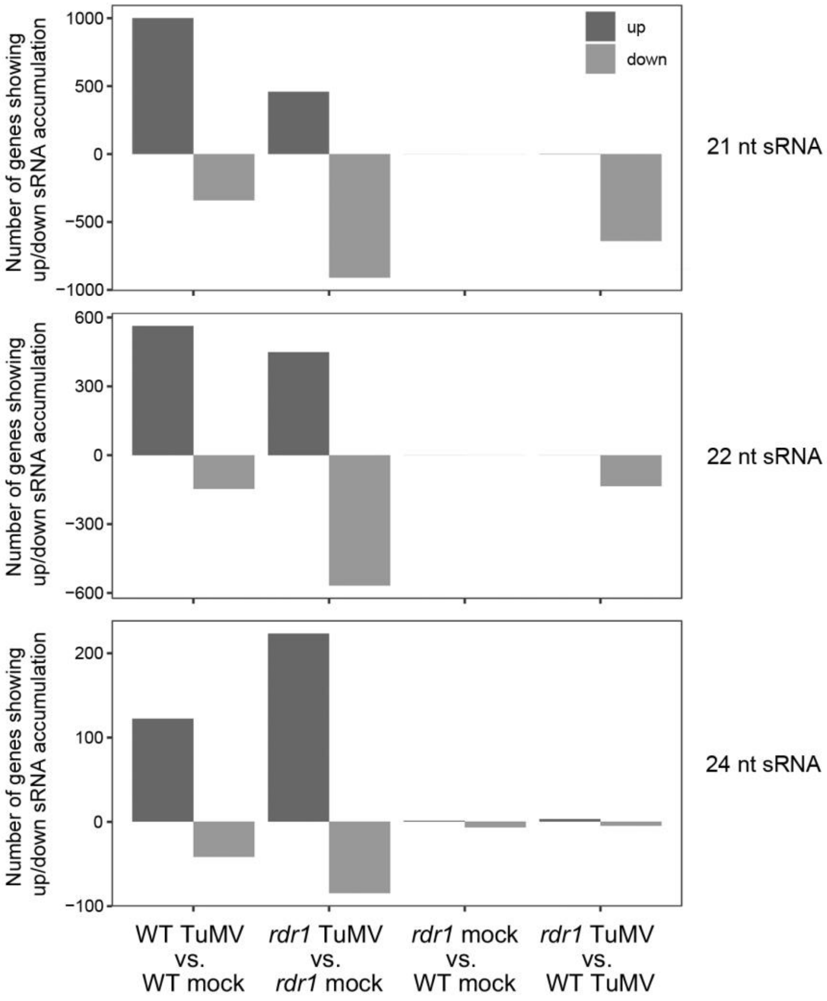

Figure S5: Plots showing two-fold increased (up) or decreased (down) accumulation of sRNA mapping to the Arabidopsis genome in mock- and TuMV-infected flower apices of WT and *rdr1* plants, in pairwise comparisons indicated on the bottom horizontal axis. On the vertical axis the plots display the number of Arabidopsis genes from which the up- or down-regulated sRNA are derived. The plots have been divided according to sRNA size: 21nt (top), 22nt (middle) and 24nt (bottom).

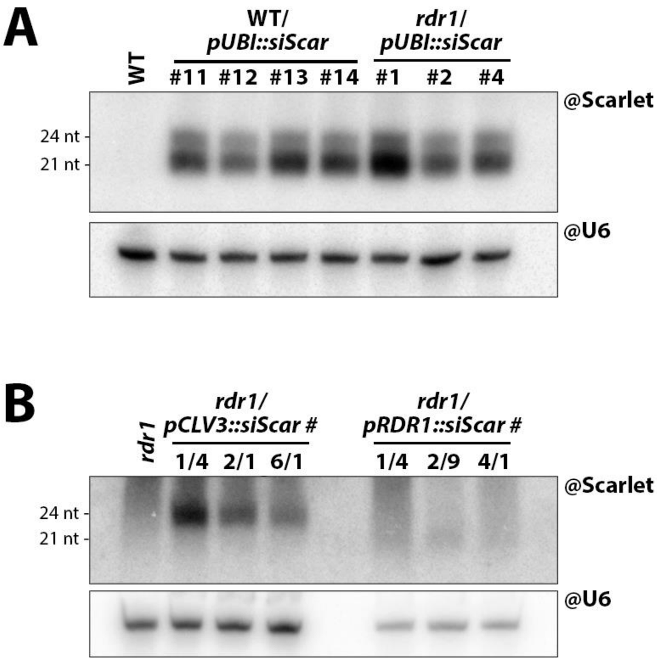

Figure S6: (A) PAGE northern blot detection of Scarlet-derived sRNA in seedlings of WT and *rdr1* transgenic lines expressing the *siScar* hairpin construct under the control of the *pUBI* promoter. Each sample is an independent transgenic line. snRNA U6 is used as loading control. **(B)** As in (A), but in flower apices (meristem and small flower buds) of *rdr1* lines expressing *siScar* through the *pCLV3* promoter (left) and seedlings expressing it through the *pRDR1* promoter. Of note, the *pCLV3*-driven hairpin is processed only into 24 nt-long siRNA.

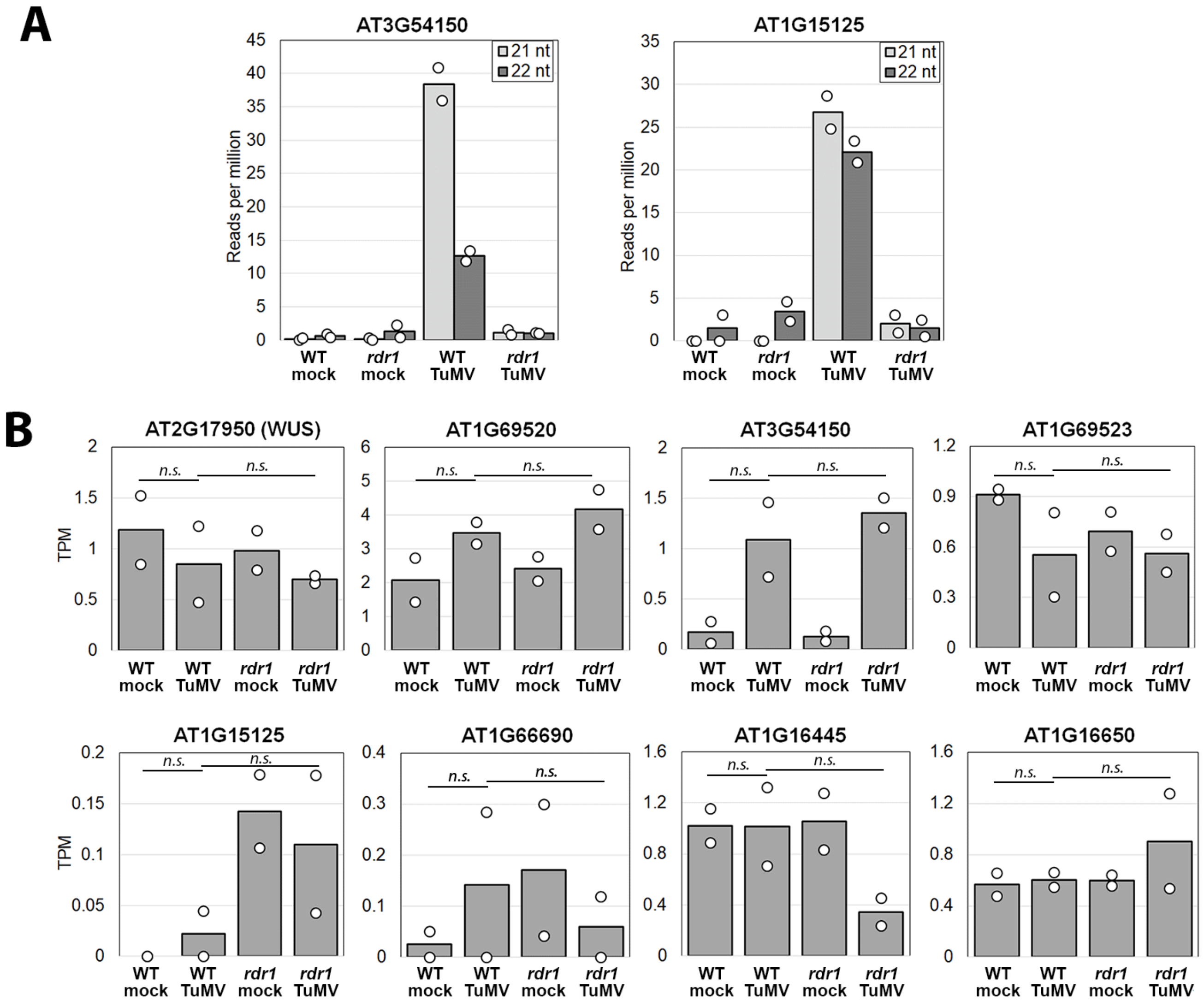

Figure S7: (A) Reads per million of small RNA of 21 or 22 nt derived from two methyltransferase genes indicated above, assessed by sRNA sequencing. For both sRNA clusters, WT TuMV vs. WT mock and *rdr1* TuMV vs. WT TuMV pairwise comparisons revealed significant differences with adjusted p value < 0.05. **(B)** Transcripts per million (TPM) of *WUS* and the methyltransferase genes indicated above, assessed by smartseq3 mRNA sequencing on the same samples used for sRNA sequencing shown in Figures S4-S5 and above. None of the WT TuMV vs. WT mock and *rdr1* TuMV vs. WT TuMV pairwise comparisons revealed differences with adjusted p value < 0.05 (*n.s.*). In (A) and (B), dots represent biological replicates.

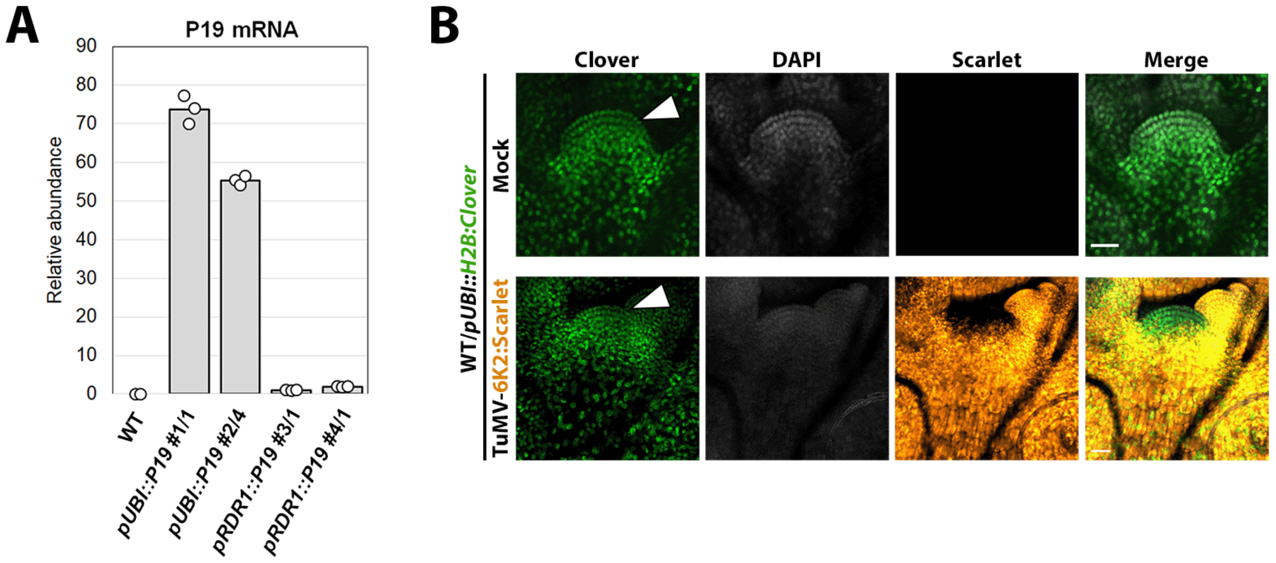

Figure S8: (A) RT-qPCR quantification of *P19* expression in upper stems and inflorences of the transgenic lines marked at the bottom, relative to *pRDR1:P19* #3/1. Dots indicate technical replicates. **(B)** Laser confocal microscopy images of meristems from mock and TuMV-6K2:Scarlet-infected plants expressing *H2B:Clover* through the *pUBI* promoter. Expression is ubiquitous, including the L1 and L2 stem cell layers (white arrowheads). DAPI fluorescence in grayscale, Scarlet in orange-to-yellow. Scale bar: 20 µm.

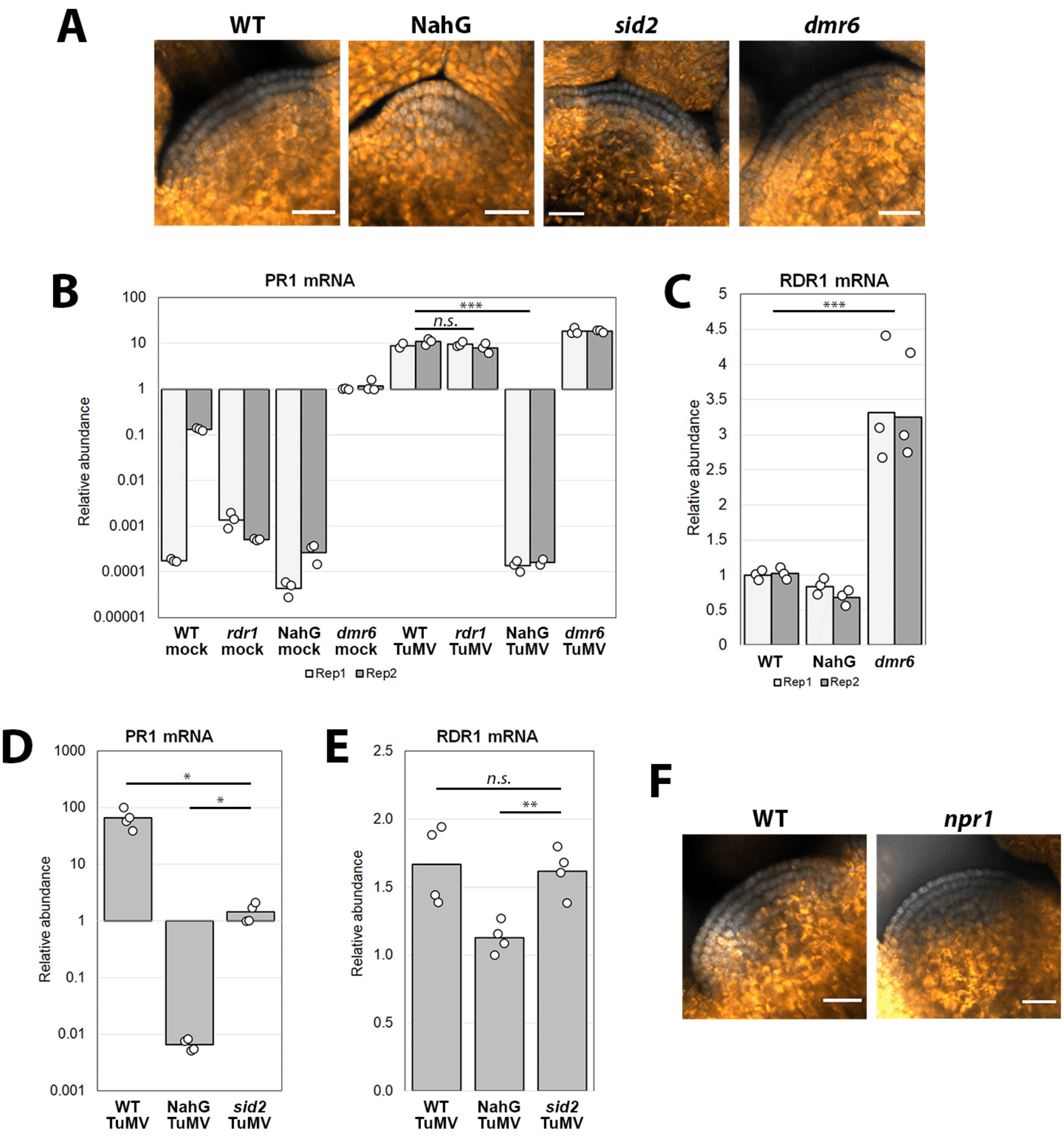

Figure S9: (A) Laser confocal microscopy images of meristems from WT, NahG, *sid2* and *dmr6* plants infected with TuMV-6K2:Scarlet. DAPI fluorescence in grayscale, Scarlet in orange-to-yellow. Scale bar: 20 µm. **(B)** RT-qPCR quantification of *PR1* expression in systemically infected tissues in the genotypes indicated after mock or TuMV-6K2:Scarlet inoculation. Each bar is a biological replicate of tissues from 4-5 plants, each dot is a technical replicate, relative abundance values are plotted on $\log_{10}$ scale. **(C)** RT-qPCR quantification of *RDR1* expression in seedlings of WT, NahG and *dmr6*. Each bar is a biological replicate of 10+ seedlings, each dot is a technical replicate. **(D)** RT-qPCR quantification of *PR1* expression in TuMV-6K2:Scarlet systemically infected tissues in the genotypes indicated. Each dot is a biological replicate of tissues from 4-5 plants, relative abundance values are plotted on $\log_{10}$ scale. **(E)** Quantification of *RDR1* mRNA in the samples described in (D), with relative abundance values plotted on a linear scale. (B), (C), (D) and (E): *n.s.*: $p>0.05$; *: $p<0.05$; **: $p<0.01$, ***: $p<0.001$. **(F)** Laser confocal microscopy images of meristems from WT and *npr1* plants infected with TuMV-6K2:Scarlet. DAPI fluorescence in grayscale, Scarlet in orange-to-yellow. Scale bar: 20 µm.

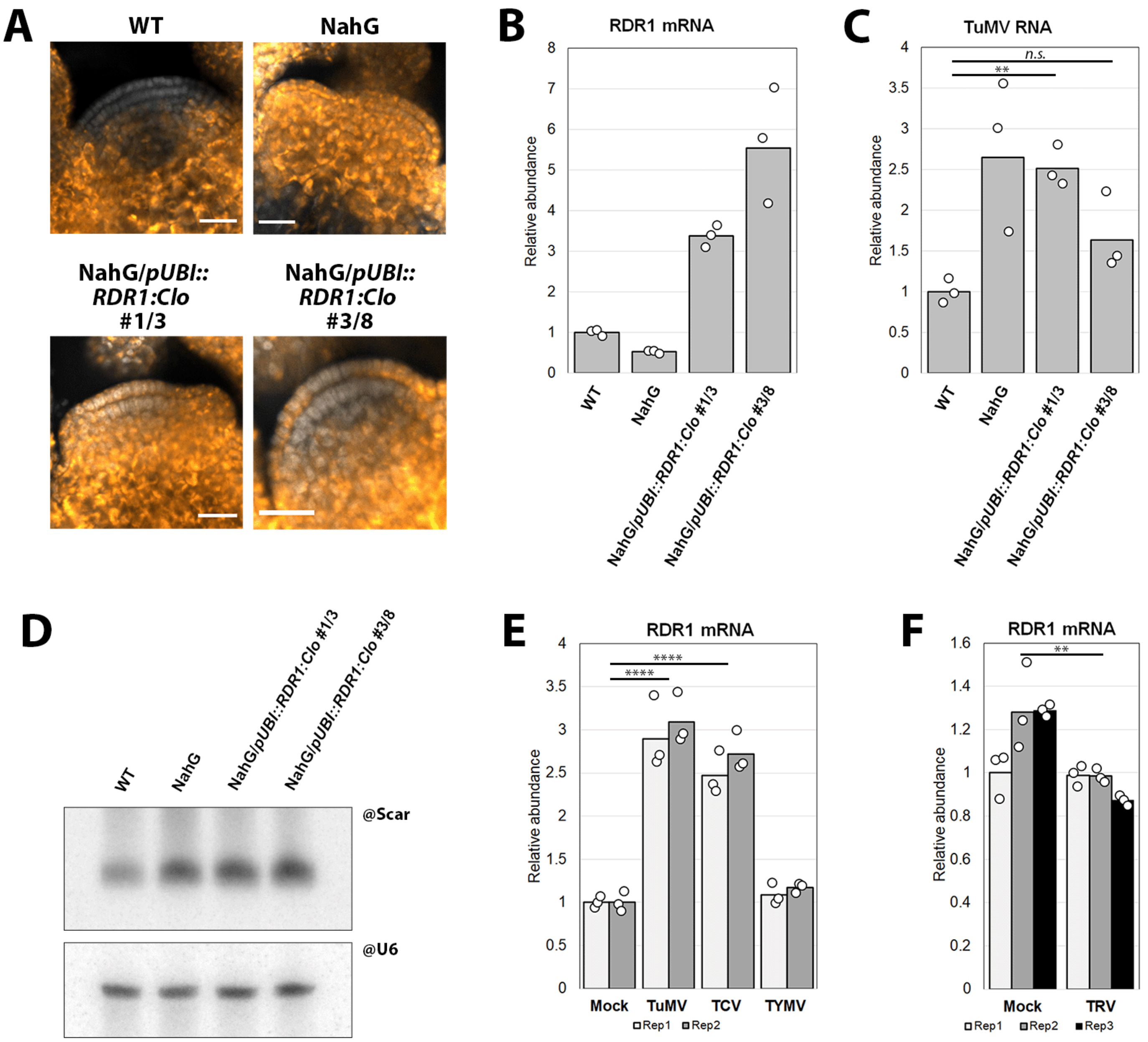

Figure S10: (A) Laser confocal microscopy images of meristems from WT, NahG, and two NahG transgenic lines expressing Clover-tagged RDR1 through the *pUBI* promoter, infected with TuMV-6K2:Scarlet. DAPI fluorescence in grayscale, Scarlet in orange-to-yellow. Scale bar: 20 µm. **(B)** RT-qPCR quantification of *RDR1* expression in the plants described in (A), in TuMV-6K2:Scarlet systemically infected tissues. Each dot is a technical replicate. **(C)** RT-qPCR quantification of TuMV gRNA accumulation in the samples in (B). **(D)** PAGE northern blot detection of Scarlet-derived sRNA in the samples in (B,C). snRNA U6 is used as loading control. **(E)** RT-qPCR quantification of *RDR1* expression in WT plants systemically infected by TuMV, TCV and TYMV. Each bar is a biological replicate of tissues from 4-5 plants, each dot is a technical replicate. **(F)** As in (E), on plants infected by TRV-Scarlet. (B), (C), (E) and (F): *n.s.*: $p>0.05$; *: $p<0.05$; **: $p<0.01$, ***: $p<0.001$, ****: $p<0.0001$.

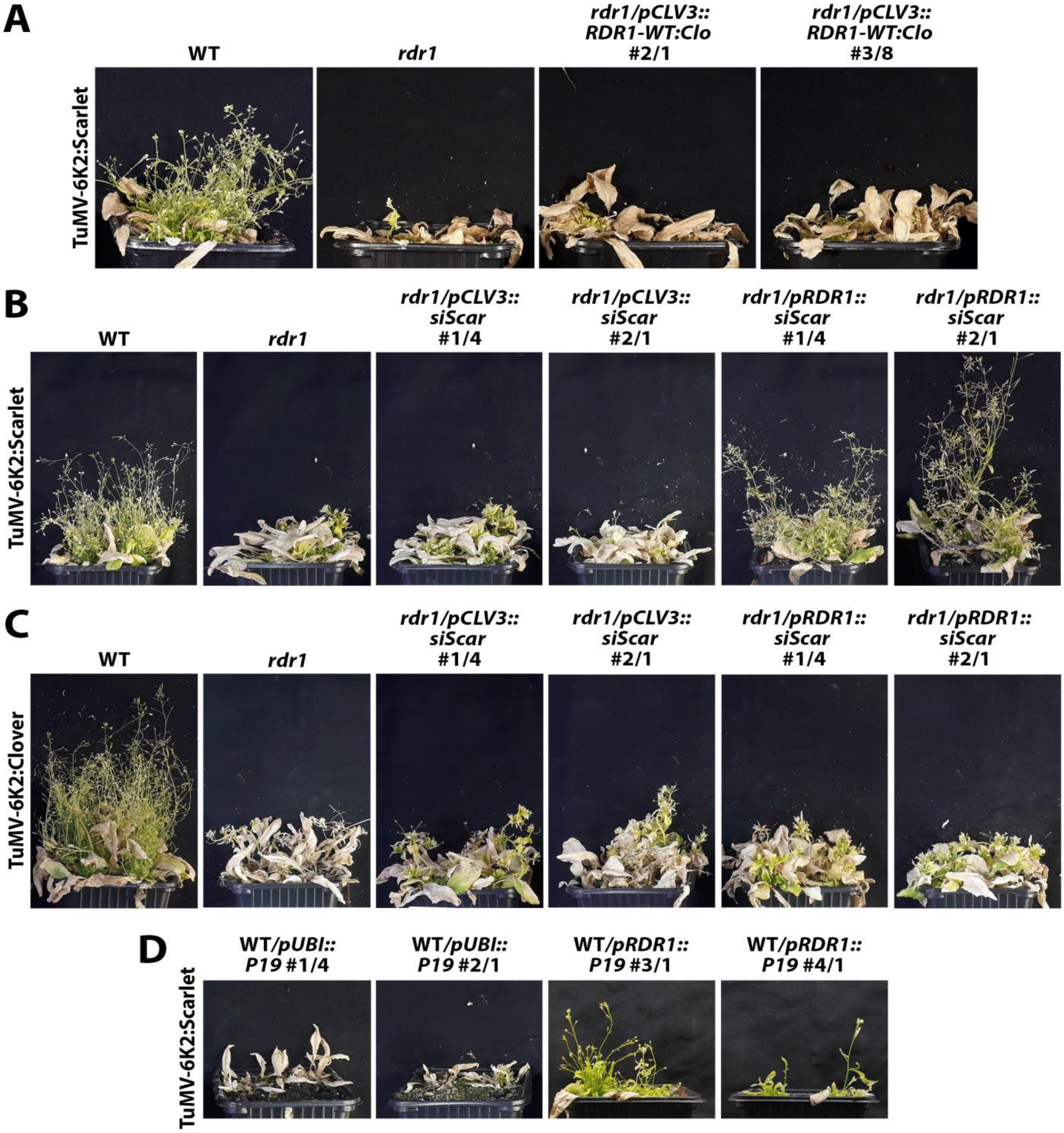

Figure S11: (A) Photos of plants one month after inoculation with TuMV-6K2:Scarlet. The two *rdr1* transgenic lines on the right express Clover-tagged RDR1 through the *pCLV3* promoter. WT and *rdr1* controls are on the left. **(B)** As in (A), with two *rdr1* transgenic lines expressing the *siScar* construct under the *pCLV3* promoter (middle) or the *pRDR1* promoter (right). **(C)** The lines described in (B), but infected with TuMV-6K2:Clover. **(D)** As in (A), of two transgenic lines expressing TBSV P19 through the *pUBI* promoter (left) and two under the *pRDR1* promoter.

Final binary vector	Destination vector	A unit	B unit	C unit	D unit	E unit	F unit
pGGSun-pCLV3::RDR1-WT:Clo-Hygro	pGGSun	pGGA-pCLV3	pGGB-Dummy	pMini-GGC-RDR1-WT	pGGD-Clover	pGGE-CLV3ter	pGGF-Hygromycin-res
pGGSun-pCLV3::siScar-Hygro	pGGSun	pGGA-pCLV3	pGGB-hpScar	pUC57-PDKintron	pGGD-hpScar-AS	pGGE-CLV3ter	pGGF-Hygromycin-res
pGGSun-pRDR1::RDR1-WT:Clo-Hygro	pGGSun	pMini-GGA-pRDR1	pGGB-Dummy	pMini-GGC-RDR1-WT	pGGD-Clover	pMini-GGE-RDR1ter	pGGF-Hygromycin-res
pGGSun-pRDR1::RDR1-D801A:Clo-Hygro	pGGSun	pMini-GGA-pRDR1	pGGB-Dummy	pMini-GGC-RDR1-D801	pGGD-Clover	pMini-GGE-RDR1ter	pGGF-Hygromycin-res
pGGSun-pRDR1::siScar-Hygro	pGGSun	pMini-GGA-pRDR1	pGGB-hpScar	pUC57-PDKintron	pGGD-hpScar-AS	pMini-GGE-RDR1ter	pGGF-Hygromycin-res
pGGSun-pRDR1::P19-YFPseed	pGGSun	pMini-GGA-pRDR1	pGGB-Dummy	pGGC-P19	pGGD-Dummy	pMini-GGE-RDR1ter	pGGF-YFP-seed-select
pGGSun-pUBIPars::siScar-Hygro	pGGSun	pMini-GGC-pUBIPars	pGGB-hpScar	pUC57-PDKintron	pGGD-hpScar-AS	pGGE-UBIter	pGGF-Hygromycin-res
pGGSun-pUBIPars::RDR1-WT:Clo-Hygro	pGGSun	pMini-GGC-pUBIPars	pGGB-Dummy	pMini-GGC-RDR1-WT	pGGD-Clover	pGGE-UBIter	pGGF-Hygromycin-res
pGGZ-pCLV3::H2B:Clover-Hygro	pGGZ003	pGGA-pCLV3	pGGB-Dummy	pGGC-H2B:Clover	pGGD-Dummy	pGGE-CLV3ter	pGGF-Hygromycin-res
pGGZ-pUBI10::H2B:Clover-Hygro	pGGZ003	pGGA-pUBI10	pGGB-Dummy	pGGC-H2B:Clover	pGGD-Dummy	pGGE-UBIter	pGGF-Hygromycin-res
pGGZ-pUBI10::P19-Basta	pGGZ003	pGGA-pUBI10	pGGB-Dummy	pGGC-P19	pGGD-Dummy	pGGE-UBIter	pGGF-Basta-res

Plasmid from Lampropoulos et al., PLOS ONE 2013

Table S1: GreenGate entry vectors used to assemble the binary vectors in the left column. Previously available vectors are highlighted in yellow.

Genotype	NASC stock number	Mutation	Genotyping primer name	Genotyping primer sequence	
rdr1-1/rdr6-15	N66112	*rdr1-1*	rdr1-1 LP<br>rdr1-1 RP<br>LB3-SAIL	GGGAAGTTGTGGATCGTCTCGACGC<br>CCAAGATTTGAGTTGGTTCAGG<br>tagcatctgaatttcataaccaatctcgatacac	
		rdr6-15	rdr6-15 LP<br>rdr6-15 RP<br>LB3-SAIL	TGAATCCATTCCTGAACAAGC<br>CAATGCAACCTCATCTTGGATG<br>tagcatctgaatttcataaccaatctcgatacac	
dcl2-1/dcl3-1/dcl4-2	N16391	*dcl2-1*	dcl2-1 LP<br>dcl2-1 RP<br>LbB1.3_SALK	ggctgagatacctcaaggtggttt<br>cctctccggaagtcttccacaatt<br>ATTTTGCCGATTTCGGAAC	
		dcl3-1	dcl3-1 LP<br>dcl3-1 RP<br>LbB1.3_SALK	gactagaagaggactcaatgcaat<br>cctgaagagcgtgaaggagtggaaa<br>ATTTTGCCGATTTCGGAAC	
		dcl4-2	dcl4-2 LP<br>dcl4-2 RP<br>GABI o8760	ggctgcacagctgatgattacaa<br>gccgctcgagatcatcagcaaaggaat<br>GGGCTACACTGAATTGGTAGCTC	
dmr6-2		*dmr6-2*	dmr6-2_geno-F<br>dmr6-2_geno-R	CACTTACGGTTTACCTGCTCATAC<br>ATTTTCTGTGTTTGTTACAGCGCG	Amplified with Q5 polymerase and digested with MseI restriction enzyme. CAPS genotyping
sid2-1		*sid2-1*	sid2-1_geno-F<br>sid2-1_geno-R	TGTCTGCAGTGAAGCTTTGG<br>CACAAACAGCTGGAGTTGGA	Amplified with Q5 polymerase and digested with MseI restriction enzyme. CAPS genotyping
npr1-1		*npr1-1*	npr1-1_F<br>npr1-1_R	CGTGTGCTCTTCATTTCGCTGT<br>GTGCGGTTCTACCTTCCAAAGTT	Amplified with Q5 polymerase and sequenced.

Table S2: Arabidopsis mutants used in this study (left) and the primers used for PCR genotyping (right)

Purpose	Primer name	Sequence	Comment
**RT-qPCR**	AtSAND-qPCR+	AACTCTATGCAGCATTTGATCCACT	
	AtSAND-qPCR-	TGATTGCATATCTTTATCGCCATC	
	GAPDH-qPCR+	TTGGTGACAACAGGTCAAGCA	
	GAPDH-qPCR-	AAACTTGTCGCTCAATGCAATC	
	PR1-qPCR+	CGTCTTTGTAGCTCTTGTAGGT	
	PR1-qPCR-	CCTGGTTGTGAACCCTTAGAT	
	TuMV-qPCR+	ACTTGGCGCGAATGTTGAAG	
	TuMV-qPCR-	TGAAAGCCGACCAAAACCTG	
	RDR1-qPCR+	GTGCAGTATTCAGATCCCATGA	
	RDR1-qPCR-	GACATTGACAGCTTGAAGAACAC	
**Northern blot oligo probe**	TCV-probe	CGTACTAAATTCTGAGTGCTTGC	
	TYMV-probe	GTGGAAGTGTCCGTGATG	
	U6-probe	TCATCCTTGCGCAGGGGCCA	
**Northern blot PCR probe**	GG-TuMV-S3-Scarlet+	GGTGGTGGTCTCCTGAAGGAGGAGGAGGATCTGGAGGAGGAATGGTTAGTAAAGGTGAAGCC	*
	GG-TuMV-S3-Scarlet-	ACCACCGGTCTCGTAGACTTTATAGAGTTCATCCATACCACC	*
	GG-TuMV-S2-6K2+	GGTGGTGGTCTCCTGCCAACACCAGCGACATGAG	*
	GG-TuMV-S2-6K2-	ACCACCGGTCTCGTTCATGGGTTACGGGTTC	*
**ISH probe**	TCV-ISHprobe+	ATTGCCCTCATCAACCTTCTC	
	TCV-ISHprobe-	CTGCGTTGACGGCATG	
	TYMV-ISHprobe+	TGTTCATGAAACCCAGCAAGTT	
	TYMV-ISHprobe-	CCAGGATTGTCACTGACAGGTGG	
	TuMV-ISHprobe+	ATGGCAGCAGTTACATTCGCATC	
	TuMV-ISHprobe-	TTGACGGTGTCTTATGAAGCG	

*: Primers originally used to generate entry vectors for golden gate cloning, so contain additional adapters at 5' ends

Table S3: primers used in this study, for the purposes indicated on the left. Genotyping primers arefound in Table S2
